# Dead-reckoning elucidates fine-scale habitat use by European badgers *Meles meles*

**DOI:** 10.1186/s40317-022-00282-2

**Published:** 2022-03-10

**Authors:** E. A. Magowan, I. E. Maguire, S. Smith, S. Redpath, N. J. Marks, R. P. Wilson, F. Menzies, M. O’Hagan, D. M. Scantlebury

**Affiliations:** 1 School of Biological Sciences, Queen’s University Belfast, 19 Chlorine Gardens, Belfast, BT9 5DL Northern Ireland UK; 2 Department of Biological Sciences, Institute of Environmental Sustainability, Swansea University, Swansea, UK; 3 Department of Agriculture, Environment and Rural Affairs, Veterinary Epidemiology Unit, Belfast, UK; 4 Randox Laboratories Ltd. Crumlin, Antrim, Northern Ireland UK

**Keywords:** Dead-reckoning, Badger, Home-range, Distance travelled, Movement, Accelerometer, GPS, Logger, Tuberculosis

## Abstract

**Background:**

Recent developments in both hardware and software of animal-borne data loggers now enable large amounts of data to be collected on both animal movement and behaviour. In particular, the combined use of tri-axial accelerometers, tri-axial magnetometers and GPS loggers enables animal tracks to be elucidated using a procedure of ‘dead-reckoning’. Although this approach was first suggested 30 years ago by Wilson et al*.* (1991), surprisingly few measurements have been made in free-ranging terrestrial animals. The current study examines movements, interactions with habitat features, and home-ranges calculated from just GPS data and also from dead-reckoned data in a model terrestrial mammal, the European badger (*Meles meles*).

**Methods:**

Research was undertaken in farmland in Northern Ireland. Two badgers (one male, one female) were live-trapped and fitted with a GPS logger, a tri-axial accelerometer, and a tri-axial magnetometer. Thereafter, the badgers’ movement paths over 2 weeks were elucidated using just GPS data and GPS-enabled dead-reckoned data, respectively.

**Results:**

Badgers travelled further using data from dead-reckoned calculations than using the data from only GPS data. Whilst once-hourly GPS data could only be represented by straight-line movements between sequential points, the sub-second resolution dead-reckoned tracks were more tortuous. Although there were no differences in Minimum Convex Polygon determinations between GPS- and dead-reckoned data, Kernel Utilisation Distribution determinations of home-range size were larger using the former method. This was because dead-reckoned data more accurately described the particular parts of landscape constituting most-visited core areas, effectively narrowing the calculation of habitat use. Finally, the dead-reckoned data showed badgers spent more time near to field margins and hedges than simple GPS data would suggest.

**Conclusion:**

Significant differences emerge when analyses of habitat use and movements are compared between calculations made using just GPS data or GPS-enabled dead-reckoned data. In particular, use of dead-reckoned data showed that animals moved 2.2 times farther, had better-defined use of the habitat (revealing clear core areas), and made more use of certain habitats (field margins, hedges). Use of dead-reckoning to provide detailed accounts of animal movement and highlight the minutiae of interactions with the environment should be considered an important technique in the ecologist’s toolkit.

## Introduction

Technological advancement including the miniaturisation of animal-borne data loggers now allows intricate data to be collected on animal movement and behaviour [1–3] with animal-attached tri-axial accelerometers and tri-axial magnetometers proving to be particularly useful in obtaining information on animals that are elusive or otherwise difficult to study [4]. Importantly, these same sensors are pivotal for the process of ‘dead-reckoning’, which uses movement vectors to reconstruct animal paths in detail (e.g. [5–9]). Such resolution allows inferences to be made about the relationship between the behaviour and location, particularly if the process is GPS-enhanced [6, 10]. However, while this technique has been demonstrated in humans [11] and illustrated in animals [12], there have been relatively few attempts to quantify the movements of free-ranging terrestrial animals. Such analyses should, however, help highlight the limitations of GPS systems used alone, particularly as regards infrequent fixes and linear interpolation between them [10], as well as provide a new standard in how we can define animal interaction with various landscape features, including how animals allocate behaviour to space.

The European Badger (*Meles meles*) is a semi-fossorial nocturnal social mammal and one of the largest terrestrial carnivore species currently inhabiting the British Isles [13–15]. It is also an important animal to study because of its implication in the transmission of bovine tuberculosis (bTB) to cattle [13, 16]. However, despite many decades of research, the exact mode of bTB transmission remains unclear (e.g. [17]). Indeed, knowledge of precise badger movements and behaviours, and interactions with the environment is considered important to further our understanding of potential disease transmission routes [18]. Here, we demonstrate how animal-borne GPS-enhanced dead-reckoning systems deployed on free-ranging badgers can provide information on their intricate movement paths which can then be used to acquire a better understanding of their spatial ecology, and provide particular insight with respect to the transmission of disease.

## Methods

### Study site and animals

The study was undertaken in an area of mixed arable and grazing farmland within an approximate 100 km^2^ area in rural Co. Down, Northern Ireland, near the town of Banbridge, using animals that were captured during the ‘Test and Vaccinate or Remove’ (TVR) project [19, 20]. The area is associated with persistent bTB cattle breakdowns [21]. The landscape comprises, predominantly, of improved grassland, with fields enclosed by hedges [22]. Prior to animal trapping, areas were surveyed for signs of badgers, which included badger main setts (burrow systems), outlier setts, latrines, crossing points, paths and areas of soil that had been excavated. Thereafter, traps were pre-baited for approximately seven days, and then set. For this study, two badgers, an adult male (9.67 kg) and an adult female (8.64 kg) were captured and anaesthetised [19]. Individuals were fitted with a neck collar containing a GPS logger (Tellus Light, Followit, Sweden) which was programmed to record positional fixes every 60 min between 21:00 and 04:00, which coincided with their most active periods. Neck collars were also fitted with a ‘daily diary’ (‘DD’) logger (Wildbyte-technologies, Swansea, UK) which contained a temperature sensor, a tri-axial magnetometer and a tri-axial accelerometer, programmed to record continuously at 7, 40 and 40 Hz, respectively, on each channel [23]. Loggers were encapsulated within 3D printed styrene plastic cases, each with a 3.6 V battery (1/2 AA 3.6 V 1200 mAh Lithium Thionyl Chloride, Saft, Levallois-Perret, France) that was secured to the collar that contained the GPS (total weight c. 270 g). After collars were deployed, badgers were returned to the trap at the point of capture until they had sufficiently recovered from the anaesthetic before being released. Both animals were recaptured after one week, by re-baiting the traps at the original locations. Thereafter, the DD loggers were removed, and data were downloaded. GPS, accelerometer, and magnetometer data were used to reconstruct the badger movement paths using dead-reckoning (below in brief, for detailed description of the calculations involved see [9]).

### Sett use

**Fig. 1:**
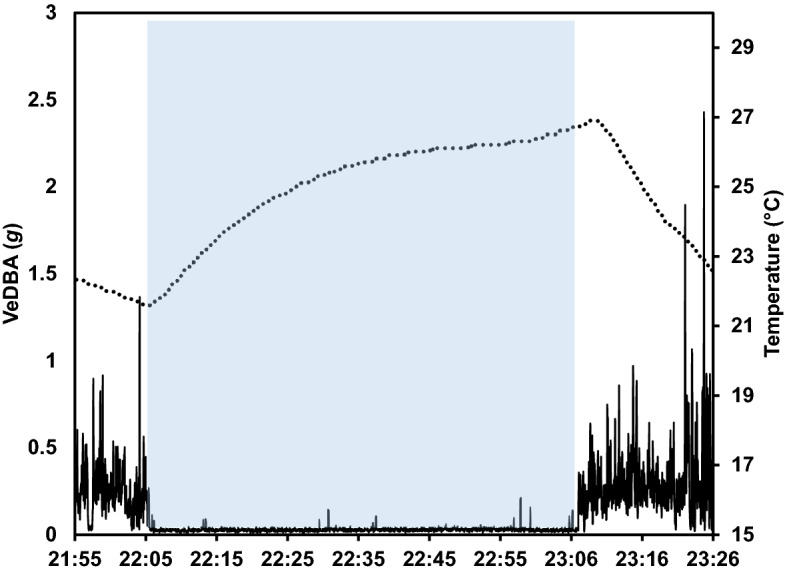
Activity measured as vectorial dynamic body acceleration (VeDBA, *g*) and ambient temperature (^o^C) measured when a badger was outside and inside its sett. The badger entered the sett at approximately 22:05 and exited again at 23:06. The shaded area represents period the badger spent inside the sett. The solid line indicates VeDBA and the dashed line indicates ambient temperature. Note the slight delay in the temperature sensor recording lower values when the animal exited the sett

**Fig. 2:**
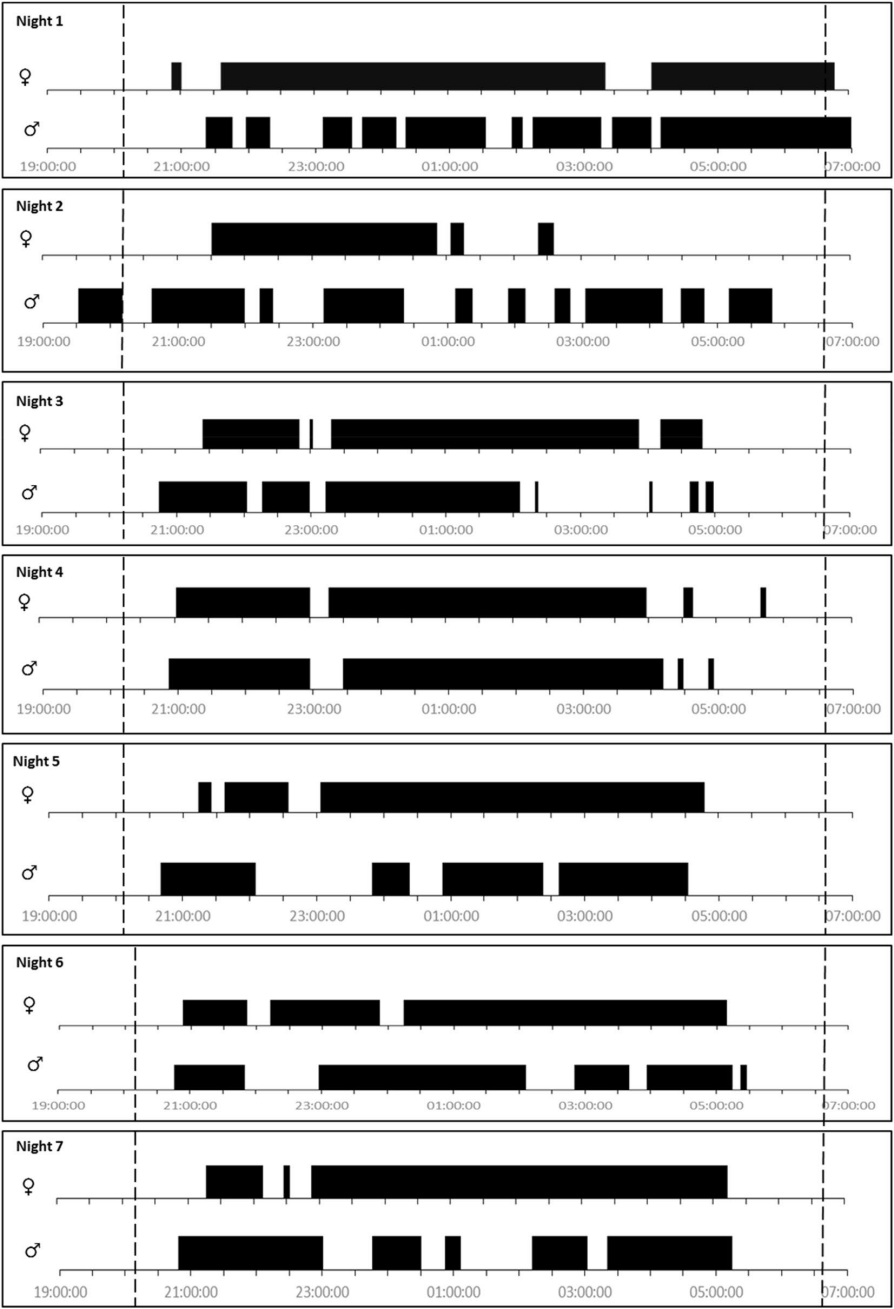
Periods spent outside the sett represented by black bars for a female (top) and male (lower) badger for 7 days after deployment of loggers

Before dead-reckoned paths could be determined, it was necessary to establish when badgers were underground within their sett, and therefore to ensure that reconstructed tracks were representative of the badger’s movements outside the sett. This was achieved using acceleration and temperature measurements. For example, Vectorial Dynamic Body Acceleration, ‘VeDBA’, [24] became dampened immediately when an animal moved underground (e.g. from c. 0.25* g* to < 0.1* g*), which was presumably indicative of restricted movements within the confines of the sett tunnels, and temperature measurements increased by about 4 °C over the period of 30 min (Fig. 1). Conversely, VeDBA values increased, and temperatures fell when animals emerged from their setts. For example, on one night, within 30 min of entering the sett, temperature measurements rose from 21.6 to 25.6 °C. This approach allowed us to establish when individuals emerged at the beginning of the night (e.g. typically at around 21:00), when they intermittently went below ground during the night, and when they returned to the sett at the end of the night (e.g. typically at 05:00) (Fig. 2).

### Dead-reckoning

**Fig. 3:**
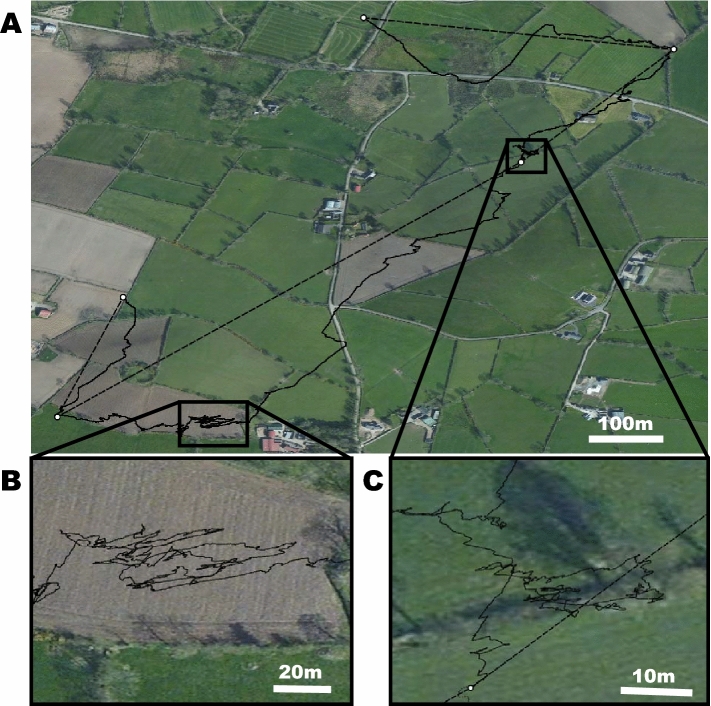
**A** Example of a GPS-enabled dead-reckoned movement path by a female badger over the course of a nightly outing. **B**, **C** Show expanded sections illustrating movements when the badger visited a field and interacted with a hedgerow, respectively. GPS fixes are shown as white dots. Straight-line movements between GPS fixes are shown by dashed lines. The GPS-enabled dead-reckoned path is shown by a black continuous line

For each night, between the hours of 21:00 and 04:00 when badgers were out of the sett, GPS and DD data were used to reconstruct dead-reckoned paths [6, 9] (Fig. 3). Vectorial Dynamic Body Acceleration was calculated as a proxy of speed [9, 10, 12] and Framework 4 software ([12], *cf*. [9]) was used to integrate hourly GPS fixes with the DD data. For this, calculations of an initial dead-reckoned track without reference to the GPS data were produced, using a nominal value for the gradient between VeDBA and speed. Then, using the time reference between the DD and the GPS, points within the GPS tracks were allocated to the time-matched points within the first iteration of the dead-reckoned tracks before the speed ~ VeDBA gradient was altered so that the positions of both systems accorded.

### Home-range and land use

Home-ranges were calculated for both badgers using the package ‘adehabitatHR’ in R (version 3.4.3) [25]. Home-ranges were calculated by two means; (i) using just the hourly GPS data and (ii) using the GPS-corrected dead-reckoned locational fixes (with positions calculated at 40 Hz). Omission of the outermost 5% of locations was undertaken to remove outliers and location errors [26]. Two estimates of home-range were then determined. These were 95% kernel utilisation distribution (KD95), and 95% Minimum Convex Polygons (MCP95) (cf. [27]). The home-ranges of each animal, calculated from GPS and 'GPS-corrected dead-reckoned data' data, for both MCP95 and KD95, were exported as polygon vector shapefiles, transferred into QGIS 3.6.3 [28], and the area of each animal’s home-range calculated in km^2^ using QGIS.

**Fig. 4:**
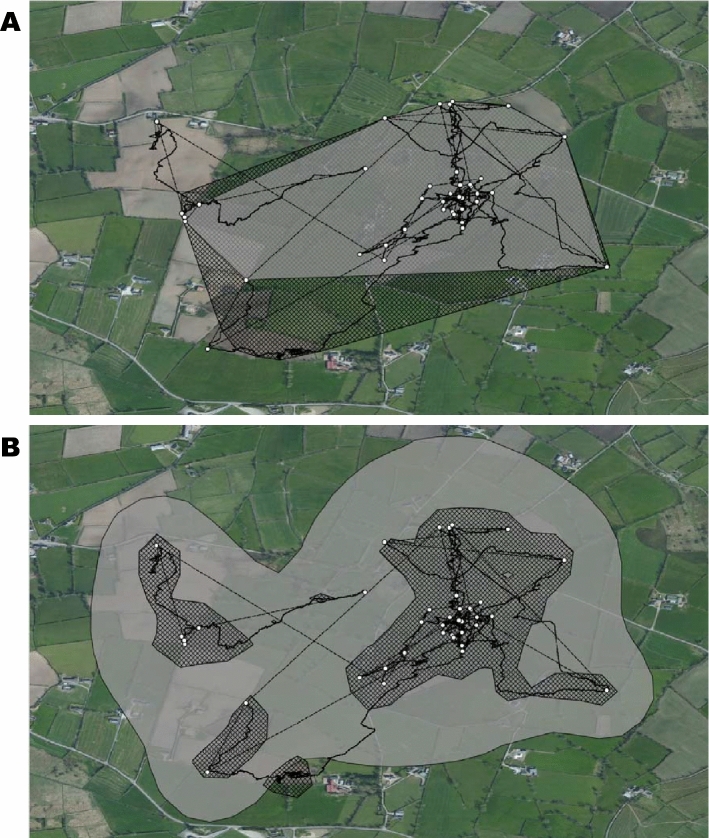
Movement paths and home-ranges of a female badger, measured using GPS (white circles) and GPS-enabled dead-reckoning (solid black lines), over seven consecutive nights of activity. Panel A shows the home-range calculated using the 95% minimum convex polygon method (MCP95) and panel B shows the home-range calculated using the 95% kernel utilisation distribution method (KD95). Grey shaded area denotes the home-range calculated using just the hourly GPS data. The black cross-hatched area indicates the home-range calculated using the GPS-enabled dead-reckoned data at 40 Hz

In addition to home-ranges, for each night that badger activity was recorded, the movement paths of both badgers were determined using both the hourly GPS data and the GPS-enabled dead-reckoned positional fixes. These fixes were overlaid onto 30 cm Digital Globe satellite imagery (Fig. 4) imported into QGIS via the OpenLayers Plugin [29]). We delineated four identifiable landscape features (fields, hedges, buildings, roads) (cf. [30]) from the Digital Globe satellite imagery by hand-drawing polygons representing individual features onto a newly created vector dataset. We then examined the times that both badgers spent in proximity to these landscape features. We considered a badger to be ‘in proximity’ of a feature if it was within 20 m of that particular feature [31]. This allowed for potential inaccuracies of the GPS loggers [32] and the effects of those features on badger behaviour [33]. The number of GPS fixes within 20 m of a particular landscape feature were summed and assumed to be proportional to the length of time spent there (e.g. for GPS locations recorded once per hour, each locational fix was assumed to record 1 h at that point). For the dead-reckoned data, the number of location points within 20 m proximity of various features was also summed and then divided by the sample rate (40 Hz) to provide the time (in seconds) within that landscape feature. We were therefore able to compare the proportion of time each night that badgers spent within 20 m of habitat features according to the GPS-enabled dead-reckoned data and GPS data. All GIS analyses were performed using the UTM Zone 29 N projection.

### Track length

To determine the distances travelled each night, by each badger, the straight-line distance between consecutive co-ordinates were calculated for both the GPS (hourly) and dead-reckoned (40 Hz) data. For this, the GPS and GPS-enabled dead-reckoned co-ordinates were uploaded into R (version 3.4.3) and the geodesic distance (the shortest distance between two points on a curved surface [i.e. the Earth]) between consecutive co-ordinates [34] calculated before summing all data.

### Statistical analyses

For path length, home-range areas and land use, differences in measurements between GPS and GPS-enabled dead-reckoned data were assessed using paired *t*-tests. Differences in path length and land use between badgers were assessed using Welch’s *t*-tests, for both GPS and GPS-enabled dead-reckoned data. Distances are reported ± standard deviations. The proportion of time spent near buildings and roads were linearly transformed by adding a constant of 1.0 to account for values equal to zero, to allow log_10_-transformation to achieve normality prior to analysis [35]. Differences in landscape use were assessed using linear models for both GPS and GPS-enabled dead-reckoned data. Here, the time that badgers spent near landscape features was included as the dependent variable whilst the landscape feature was included as the independent variable. Post hoc pairwise comparisons of time spent near each landscape feature was carried out using a Tukey honestly significant difference (HSD) test. Statistical analyses were evaluated to a 0.05 *p*-value level. All data analyses were performed in R (version 3.4.3) [25].

## Results

### Sett use

Badgers were predominantly nocturnal, emerging from the sett close to sunset (mean first emergence time = 20:12) and returning close to sunrise (mean last sett return time = 06:33) (Fig. 2). The time the badgers spent above ground, outside their sett, varied from 4 h 54 min to 10 h 22 min.

### Calculated path lengths using GPS and dead-reckoning data

There was a significant difference in calculated path distances travelled between GPS data and GPS-enabled dead-reckoned data, with badgers measured to travel more than twice as far when using the GPS-enabled dead-reckoned data than with only GPS data (2.53 ± 1.79 and 1.14 ± 0.75 km, respectively, *t*_1,13_ = 4.76, *p* < 0.001, Fig. 4). The path lengths between the two individual badgers did not differ, either when they were calculated using the GPS (0.96 ± 0.59 and 1.32 ± 0.89 km; *t*_1,10.43_ = 0.88, *p* = 0.40) or the GPS-enabled dead-reckoned (2.10 ± 1.33 and 2.96 ± 2.19 km; *t*_1,9.87_ = 0.89, *p* = 0.39) data.

### Home-range area

There was no significant difference in home-range size of each badger when calculated using the MCP95 method between GPS and GPS-enabled dead-reckoned data (*t*_1,1_ = 1.16, *p* = 0.45). Home-range areas calculated for both individuals using MCP95 were 0.38 ± 0.08 km^2^ and 0.59 ± 0.18 km^2^ for GPS and GPS-enabled dead-reckoned data, respectively. However, home-range sizes using the KD95 method for both individuals together were significantly larger when calculated using the GPS data than the dead-reckoned data (1.15 ± 0.16 km^2^ and 0.26 ± 0.09 km^2^, respectively, t_1,1_ = 17.8, *p* = 0.04, Fig. 4).

### Land use

#### Differences in land use assessed using both GPS and GPS-enabled dead-reckoned data

**Fig. 5:**
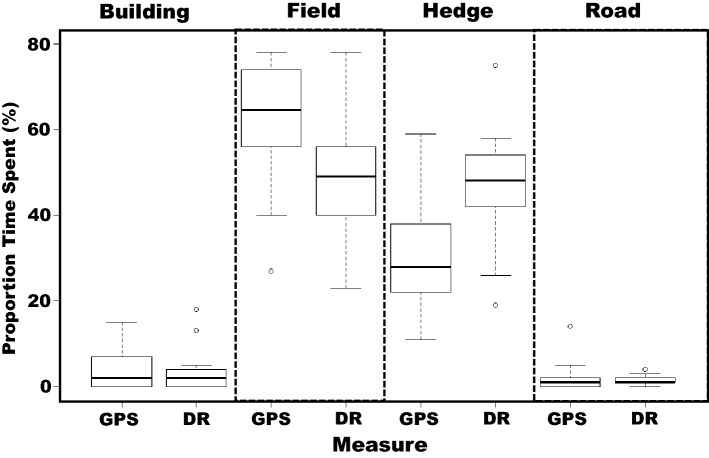
Proportion of time spent within various landscape features by two badgers according to whether the positional data were derived from GPS or GPS-enabled dead-reckoned data, labelled “GPS” and “DR”, respectively

Using just the GPS data, significant differences were noted in the times badgers spent near various landscape features (*F*_3, 52_ = 105.10, *p* < 0.001) (Fig. 5). Post hoc analyses revealed that badgers spend most of their time within fields, followed by hedges, and least time near buildings or roads. By comparison, analysis of the GPS-enabled dead-reckoned data produced different results; badgers differed in the time they spent within various landscape features (*F*_3,52_ = 122.11, *p* < 0.001), in this case, a similar amount of time was spent within fields and near hedges, and less time spent near roads and buildings.

#### Comparison of land use between GPS and GPS-enabled dead-reckoned data

Significant differences were noted in the proportions of time badgers spent within fields (*t*_1,13_ = 2.43, *p* = 0.03), and in the proportion of time spent near hedges (*t*_1,13_ = 3.05, *p* = 0.009) when the times within these habitats compared using GPS and GPS-enabled dead-reckoned data. Use of GPS-enabled dead-reckoned data revealed that badgers spent less time in fields and more time close to hedges, than did use of GPS data (Fig. 5). There were no significant differences in the calculated proportions of time spent close to buildings (*t*_1,13_ = 0.45, *p* = 0.66) or close to roads (*t*_1,13_ = 0.75, *p* = 0.47), when these were measured using data from GPS and GPS-enabled dead-reckoning, respectively.

#### Differences in land use between individual badgers

**Fig. 6:**
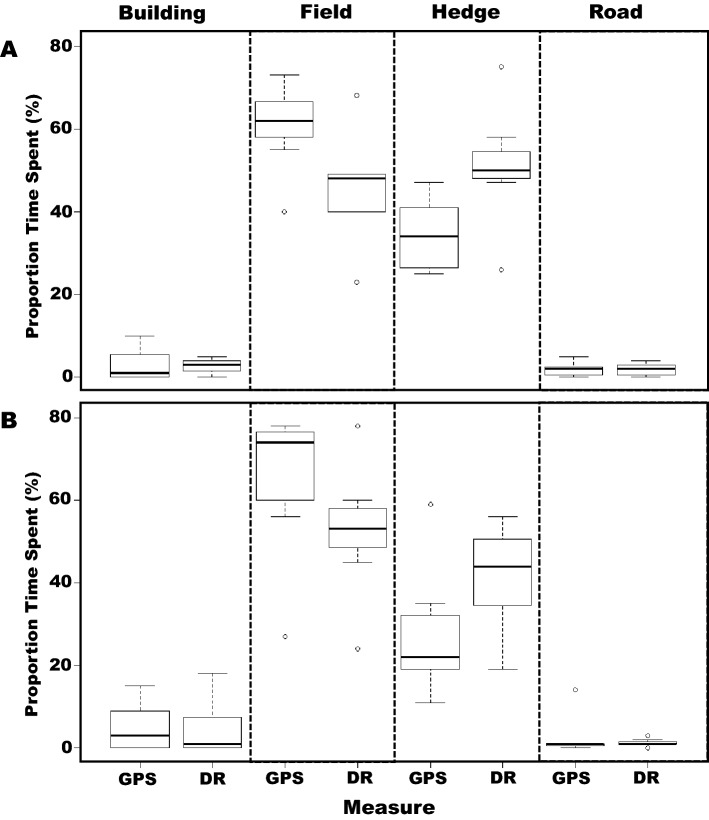
Proportion of time spent within various landscape features by two different badgers (top panel A = female; lower panel B = male) according to whether the positional data were derived from GPS or GPS-enabled dead-reckoned data, labelled “GPS” and “DR”, respectively

There were no significant differences in the proportions of time spent within fields (*t*_1,9.62_ = 0.50, *p* = 0.63), near to hedges (*t*_1,9.46_ = 0.98, *p* = 0.35), buildings (*t*_1,11.66_ = − 0.39, *p* = 0.70) or roads (*t*_1,11.66_ = 0.20, *p* = 0.84) between the two badgers when calculated using just the GPS data (Fig. 6). Similarly, there were no significant differences in the proportions of time spent within fields (*t*_1,11.62_ = 0.91, *p* = 0.38), near to hedges (*t*_1,11.95_ = 1.27, *p* = 0.23), buildings (*t*_1,8.86_ = 0.22, *p* = 0.83) or roads (*t*_1,10.15_ = 0.40, *p* = 0.70) between the two badgers when calculated using GPS-enabled dead-reckoned data.

## Discussion

The vectorial process inherent within dead-reckoning can provide detailed information on an animal’s location between sequentially collected GPS points [6] so that, dead-reckoned errors [6, 7, 36] can be corrected to provide information on animal movements with respect to their environment [9, 10]. Although this method was initially employed to deduce the movements of elusive marine species where location measurements are sporadic, and when ground-truthing can generally only occur when the animal surfaces [5, 37], this approach has great potential for the terrestrial environment [6, 38] where periodic ground-truthing using GPS location co-ordinates is less problematic [12, 39]. Nevertheless, current application of this technique to determine movement paths of free-ranging terrestrial species is surprisingly limited ([7, 9, 39] cf. semiaquatic beavers [40]).

The purpose of our study was to demonstrate the extent to which data on the movement paths and habitat use of an important but elusive terrestrial carnivore, the Eurasian badger, depend on whether animal position over time is determined by GPS or GPS-enabled dead-reckoning. Given that our study only elucidates the movements of two free-roaming badgers within a rural mixed arable and pasture farming area of Northern Ireland, we obviously cannot allude to population-level behaviours. Nevertheless, even with our small sample size, a number of important issues emerge. In particular, these issues relate to the extent to which well-defined movements of badgers, including their interactions between various habitat features such as farm buildings and livestock pasture, might elucidate their potential to transmit bovine tuberculosis to cattle [13, 16]. Previous studies have examined badger movements using GPS loggers to measured aspects such as distances travelled, home-ranges and landscape use (e.g. [18, 41, 42]). We note in this, that both our GPS and GPS-enabled dead-reckoning approaches highlighted the general importance of fields, presumed useful for foraging [18, 43], and that both approaches ascribed little time spent near roads or buildings (Fig. 4), which accords with observations made by Mullen et al*.* [44] and Campbell et al*.* [16]. However, our work also indicates that such GPS-based estimates may need to be modified for a more comprehensive picture of badger movement ecology. This is manifest by our study that reveals, for instance, that GPS-enabled dead-reckoned data provides greater estimates of distances travelled, in this case *c*.2.2 times longer, than GPS data alone, an obvious consequence of determining distance in a more tortuous path [6]. At face value, assuming that badgers have potential to transmit disease as a function of the area traversed (simplistically, this being given by the path length multiplied by some nominal ‘infection width’), this means that GPS-enabled dead-reckoning ascribes a probability of disease transmission that is proportionately higher than that deduced from GPS data alone. But, this difference is perhaps less important than space-specific patterns that come from GPS-enabled dead-reckoning compared to just GPS. A good example of this is the finding that GPS-enabled dead-reckoned badgers spent proportionately less time within fields and more near hedgerows (Fig. 4) than the GPS data alone. This is particularly relevant given that hedgerows constitute a much smaller proportion of the total area and so, all things being equal, would be expected to have a correspondingly small time allocation. Hedges and boundaries may, in fact, be areas where badger setts are located, where the animals forage, or where they form latrines [45]. All of this is relevant because badger excreta may be concentrated within such areas [46], again increasing the risk of disease spread within and between such highly utilised sites [47]. In fact, the specifics provided by GPS-enhanced dead-reckoned data should make it possible to provide a detailed probabilistic space–time use approach for both badgers and cattle, if the livestock are similarly tagged (*cf*. [39]). We envisage a modelling approach that could create a badger area density map incorporating a time element so that both absolute density and the potential for disease transmission decaying over time since the last time the area was visited by the different individuals [48] to provide pivotal information regarding disease transmission. Livestock space and time use of the area could be superimposed on this to help elucidate the probability of cross-species infection [49]. We note that a similar approach has been adopted for the Covid-19 crisis, with a major part of the predictive capacity of transmission being the accurate definition of space use by individuals over time [50]. At a coarser scale, all this makes effective calculation of home-range more than just academic. We noted appreciable differences in apparent habitat utilisation between GPS and GPS-enhanced dead-reckoned data with badger home-ranges calculated using MCP95 from GPS data being 57% smaller. Conversely, home-ranges calculated using KD95 with GPS data were 78% larger than those calculated with the GPS-enhanced dead-reckoned data (Fig. 6). These discrepancies occur because the GPS-enhanced dead-reckoned data produces a larger polygon for the MCP95 because the home-range estimates include internal areas which animals do not necessarily use (e.g. the centre of the field if the badger only walked around the periphery, which is only likely to be shown by GPS-enhanced dead-reckoning). In contrast, KD95 provide home-range estimates based on the clustering of co-ordinates, and the higher frequency provided by GPS-enhanced dead-reckoning provides a tighter clustering of points where the badgers had been, excluding unused areas and producing smaller home-range areas.

## Conclusions

Our results, although only derived from two animals over a total of 14 badger days, clearly show the advantages of GPS-enhanced dead-reckoning compared to temporally coarser position-determining systems such as GPS for examining factors important in disease transmission. Although we note that it is possible to derive GPS positions at, e.g. 1 Hz, this necessitates a substantial power source [7] and also does not guarantee the accuracy of the GPS, which is affected by landscape features such as mountains and trees [51, 52]. We also note that such an approach should help elucidate the extent to which culling badgers, in an effort to reduce bTB transmission, may affect the behaviour of conspecifics [53–55]. Indeed, the ability to describe in detail what animals do in relation to environmental circumstance must be considered a pivotal point in predicting the spread of any transmittable disease and GPS-enhanced dead-reckoning seems a powerful way of moving closer to this goal.

## Data Availability

The datasets used and/or analysed during the current study are available from the corresponding author on reasonable request.
